# Left Atrial Appendage Aneurysm: A Case Report and Literature Review

**DOI:** 10.7759/cureus.56280

**Published:** 2024-03-16

**Authors:** Juan D Ayala Torres, Jorge Alejandro Sepulveda Gallego, Mateo Gonzalez Gonzalez

**Affiliations:** 1 Radiology, Universidad de Antioquia, Medellín, COL; 2 Radiology, Hospital Universitario San Vicente Fundación, Medellín, COL

**Keywords:** case report, echocardiogram, aneurysectomy, diagnostic imaging, left atrial appendage

## Abstract

The left atrial appendage aneurysm is an uncommon condition that has garnered attention from the medical community due to its low incidence and varied clinical manifestations. The difficulty in identification is reflected in its incidental detection in imaging studies such as echocardiograms and tomographies, while symptoms range from mild to severe, including heart failure and thromboembolic events. The complex etiology includes congenital and acquired factors, and its management focuses on preventing complications through surgical resection, accompanied by medical strategies such as controlling heart rhythm and anticoagulation.

The case of a 67-year-old woman with significant medical history illustrates these challenges. Despite an inconclusive initial diagnosis, a tomography revealed an aneurysm with an intracavitary thrombus, leading to successful surgical resection. However, subsequent infectious complications resulted in her death.

The average age of diagnosis is around 30 years, and while it is more common in women, there are no significant gender differences. Surgical management remains the preferred option, especially in severe cases, although in some patients, a watchful waiting approach is chosen.

In conclusion, the left atrial appendage aneurysm is a complex entity that requires a multidisciplinary approach to improve clinical outcomes. Early diagnosis and appropriate treatment are crucial to prevent serious complications and improve the quality of life of affected patients.

## Introduction

Left atrial appendage (LAA) aneurysm is a rare condition that has attracted medical attention due to its low incidence and diverse clinical presentations. Since it was first described by Dimond et al. in 1960 [1], to date approximately 100 cases have been reported. This cardiovascular disorder poses diagnostic and therapeutic challenges due to its infrequent nature and the limited available literature [2,3]. Diagnosis is mainly incidental and through intraoperative findings, autopsies, and imaging studies such as echocardiography, tomography, and resonance, which allow differentiating between cardiovascular structures of the mediastinum. The LAA aneurysm presents a wide spectrum of clinical manifestations, ranging from asymptomatic cases to signs derived from the compression of adjacent structures, heart failure, arrhythmias, thromboembolic events, and sudden death [2-6]. They are classified based on their etiology into congenital and acquired, with congenital ones being more common, such as those derived from pectinate muscle dysplasia and pericardial defects, and acquired ones resulting from structural and functional alterations due to various cardiovascular diseases such as mitral valve disease, syphilitic myocarditis, and tuberculosis, among other causes. Their location with respect to the pericardium further classifies them as intra- and extra-pericardial [2,3,6-8]. The focus in managing this condition is on preventing structural complications that can lead to heart failure, lethal arrhythmias, systemic embolism, and mediastinal compression syndromes. Surgical resection of the aneurysm constitutes the cornerstone of treatment, accompanied by medical strategies such as heart rhythm control and anticoagulation [2,6,7,9,10]. In summary, LAA aneurysm presents a significant clinical challenge that demands a deep understanding of its clinical presentation and therapeutic options. Through the presentation of this case, we aim to illustratively showcase this complex cardiovascular entity, highlighting the importance of up-to-date multidisciplinary management to improve clinical outcomes and the quality of life of affected patients.

## Case presentation

**Figure 1:**
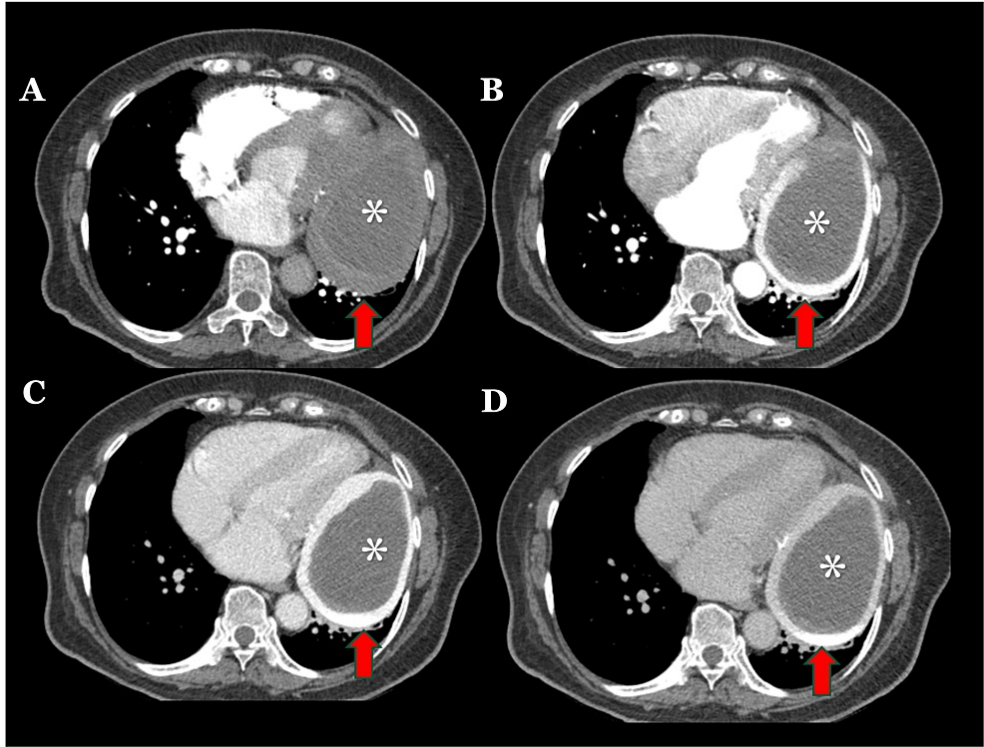
Chest angiotomography in axial projections. Multiplanar reconstruction in short-axis view in four chambers. Saccular pseudoaneurysm dependent on the left atrial appendage (red arrows) with mural calcifications, showing progressive peripheral enhancement in the dynamic contrast phases (A- D) and a central oval filling defect corresponding to an intraluminal thrombus (asterisk).

**Figure 2:**
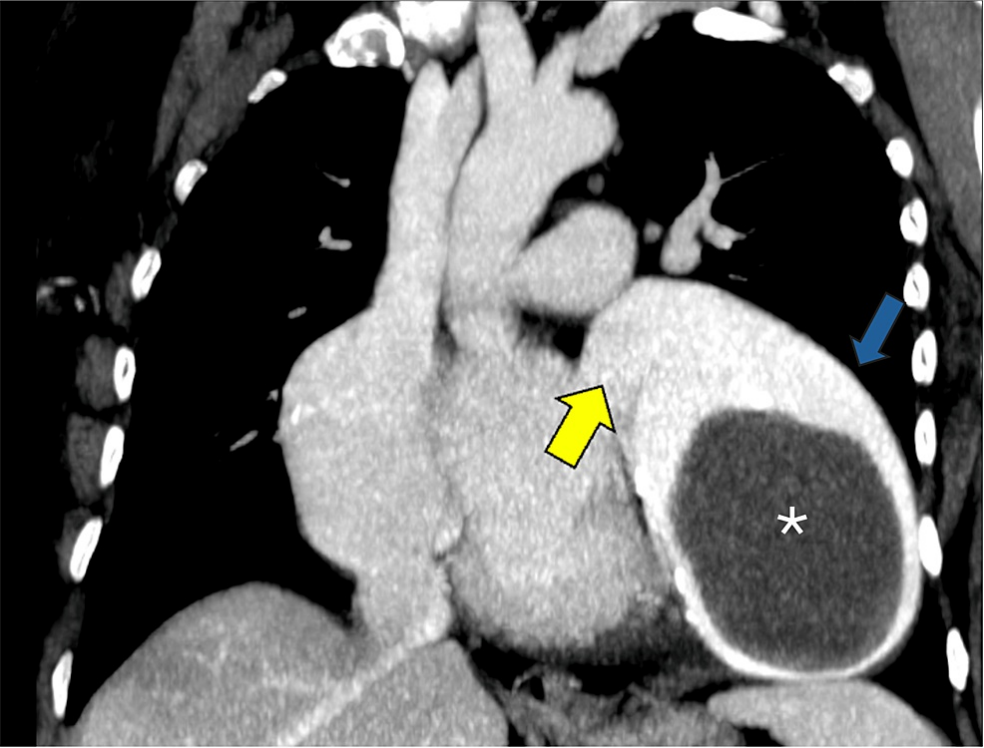
Contrast-enhanced chest tomography with maximum intensity projection in oblique coronal reconstruction Saccular aneurysm of the left atrial appendage (blue arrow) with the presence of mural thrombus inside (asterisk) can be noted. Communication with the left atrium is evident in the image (yellow arrow), as well as compression of adjacent cavities.

**Figure 3:**
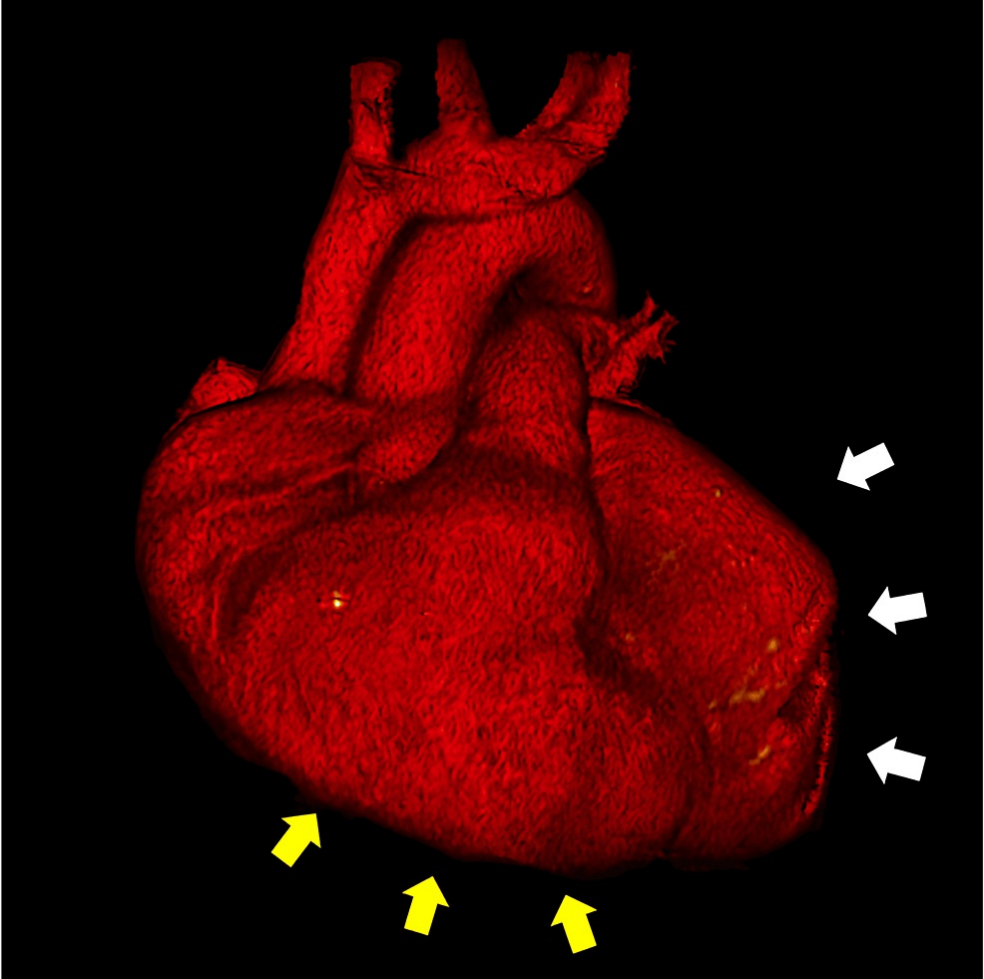
Chest angiotomography with 3D reconstruction Saccular aneurysm of the left atrial appendage (white arrows) with a size similar to that of the left ventricle (yellow arrows) can be noted.

A 67-year-old female patient residing in the city of Montería, in the Department of Cordoba, Colombia, with a history of obesity, diabetes mellitus, and arterial hypertension, presented with a progressive dyspnea and deterioration of her functional class. During her initial evaluation, a left cardiac mass was detected by echocardiography, prompting referral to a tertiary referral hospital in the city of Medellin, Antioquia. Congestive heart failure with congestive symptoms was documented, and a second echocardiogram yielded inconclusive results; therefore, a chest angiotomography (CTA) was requested by the treating physicians. The angiotomography revealed a saccular aneurysmal dilation of the LAA, located within the pericardium adjacent to the left margin of the heart, with mural calcifications and an intraluminal thrombus occupying most of its lumen (Figure 1). The aneurysm had a maximum diameter of 12 cm and was compressing the left cardiac cavities (Figures 2, 3).

Due to the size of the aneurysm and associated heart failure, the patient underwent surgery for aneurysm resection and correction of the cardiac defect, without immediate complications. Unfortunately, she passed away during her stay in the intensive care unit due to infectious complications in the context of urinary sepsis caused by Klebsiella spp.

## Discussion

Since it was first described by Dimond et al. in 1960 [1], to date, approximately 100 cases have been reported. In 1962, Parmley et al. documented two cases of LAA abnormalities associated with dilation of the left atria during a surgical procedure. [8], and, in 1968, Godwin et al. advanced diagnostic techniques by attempting to visualize an LAA aneurysm using I-131-labelled albumin, expanding possibilities beyond angiography and surgical exploration [11]. Post-mortem reports continued in subsequent decades [12].

A milestone was reached in 1982 when Foale et al. characterized an LAA aneurysm using transthoracic echocardiography (TTE) [13]; by 1983, German literature was reporting LAA aneurysm characterization using echocardiography and computed tomography [14]. Meanwhile, other European researchers were exploring the value of echocardiography and tomography in diagnosing endocavitary thrombi [15], establishing a foundation for axial imaging use in diagnosing, characterizing, and surgically planning for these patients [2]. Finally, in 1990, Frambach et al. and Comess et al. reported two cases diagnosed using TTE and transesophageal echocardiography (TEE), respectively [2,16].

The widespread availability of imaging techniques, along with advances in contrast media and synchronization protocols with cardiac rhythm, has increased the number of reports of this entity and contributed to understanding its natural history and associated morbidity and mortality [5,6,9].

LAA aneurysm has been classified as congenital or acquired, with 90% of the cases being congenital [17]. The reason is not clear, although the anomaly is probably due to congenital dysplasia of the atrial pectinate muscles [17]. Acquired LAA aneurysm is often secondary to mitral valve disease or other conditions, leading to elevated left atrial pressure [17]. The common histopathological finding of both congenital and acquired LAA aneurysm is fibrosis of the endocardium or myocardium [17] . Hypertrophied myocardium with an increase in interstitial fibrous tissue may be seen in some cases [17]. Rarely, LAA aneurysm has been reported to be associated with congenital cardiac abnormalities, with Gold et al. reporting five patients with atrial septal defect, one patient with ventricular septal defect, one with anomalous pulmonary venous drainage, one with mitral valve cleft, and one with tricuspid atresia [17].

It can manifest in individuals of any age group, with documented prenatal cases ranging from 28 weeks of gestation to 88-year-old patients. The average age of those affected is 30 ± 20 years, with most diagnoses made during the third decade of life, representing 24.8% of recorded cases. This age distribution suggests the possibility of progressive aneurysm enlargement over time [2,9,18]. There have been no significant differences observed in LAA aneurysm prevalence between men and women. However, an analysis suggests that the percentage of female cases (52.5%) is slightly higher than male cases (47.5%) [2,9]. Although 90% of LAA aneurysm cases are congenital, the vast majority are diagnosed in adulthood when symptom presentation leads to thoracic imaging studies [2,3,7,9].

Most LAA aneurysm patients undergo a chest X-ray, although tomographic studies, as well as magnetic resonance imaging, play a crucial role in diagnosing and characterizing aneurysmal lesions of the cardiac cavities. Echocardiography, initially transthoracic and then transesophageal, remains the primary imaging modality of choice [2,6,9].

Chest X-ray findings may include alterations in the left cardiac silhouette in the form of a mass, cardiomegaly, or a combination of both. However, these findings are nonspecific and require a broad differential diagnosis, ranging from left ventricular aneurysms, cysts, and mediastinal tumors, to coronary sinus dilatation and valvulopathies with secondary dilatation [3,19].

Simple chest tomography (CT) allows visualization of a left atrial mass, but the absence of contrast medium hinders its characterization, limiting the definition of its content and communication with the left cavities. Additionally, cardiac motion artifacts reduce its specificity [2,3,6,7,9].

CTA with electrocardiographic gating resolves these issues, allowing visualization of the aneurysmal sac's communication with the left atrial cavity, as well as detecting thrombi inside and associated complications. It also offers greater anatomical resolution by reducing motion artifacts, facilitating surgical planning [2,3,6,7,9].

Cardiac magnetic resonance imaging, although less available and with a longer acquisition time, offers 91% accuracy and greater temporal resolution, allowing evaluation of the aneurysm's anatomical relationships and providing detailed functional information [4,6,9].

Both TTE and TEE are essential in LAA aneurysm diagnosis [2,3,7,9,18]. Although TTE has limitations due to the acoustic window, TEE, with specific views, allows diagnostic accuracy of 83.3%. Typical echocardiography findings include a sac-like structure attached to the left atrium, with an average length exceeding 5 cm, and thrombus detection, especially with TEE [3,6,16,18].

Since the early reports, the focus on managing of the LAA aneurysm has been on preventing complications, with the surgical strategy widely accepted even in asymptomatic patients due to the risk of embolism and lethal arrhythmias [2,8,9,12].

Highly invasive approaches, such as median sternotomy, have evolved from being the only treatment option to being preferred in patients with voluminous aneurysms especially when associated with thrombi. On the other hand, in patients with smaller aneurysms and no endocavitary thrombus, left lateral thoracotomy may be considered, although it requires greater technical skills and institutional resources due to its greater complexity [9].

To date, expectant management has only been documented in 11 patients, mainly due to refusal of primary treatment, high surgical risk, or neurological contraindications due to massive embolisms and secondary brain death [9].

## Conclusions

LAA aneurysm, a clinical rarity, demands a comprehensive diagnostic approach, where echocardiography serves as the initial imaging modality of choice. Angiotomography and magnetic resonance imaging provide clarity in complex cases and allow for precise pre-surgical mapping. Surgical resection of the aneurysm is pivotal in preventing morbidity and mortality, always prioritizing the appropriate selection of the candidate for intervention. This complex entity requires active detection, as it is quintessentially an incidental diagnosis. It also necessitates a robust, multidisciplinary treatment approach to achieve the best possible clinical outcomes for each patient.
